# Differentiation, maturation, and collection of THP-1-derived dendritic cells based on a PEG hydrogel culture platform

**DOI:** 10.1007/s10529-023-03457-w

**Published:** 2024-01-17

**Authors:** Jaeho Choi, Chang Seok Ki

**Affiliations:** 1 Department of Agriculture, Forestry and Bioresources, Seoul National University, Seoul, 08826 Republic of Korea; 2 Research Institute of Agriculture and Life Science, Seoul National University, Seoul, 08826 Republic of Korea

**Keywords:** Dendritic cell, Hydrogel, PEG, 3D culture, Cell harvesting

## Abstract

**Purpose:**

Dendritic cell (DC) is a spearhead responsible for immune response and surrounded by extracellular matrix in three-dimensional (3D) tissue. Nevertheless, conventional DC culture has relied on suspension or two-dimensional (2D) tissue culture plate (TCP)-based culture system. This culture condition often fails to recapitulate the physiological behavior of DC in real tissue. In this work, the effect of culture condition on DC physiology was explored with varying 3D hydrogel property (i.e., degradability, adhesion, and stiffness). In particular, DC differentiation and maturation in 3D were evaluated comparing the conventional TCP-based culture condition.

**Method:**

THP-1 cells were encapsulated in poly(ethylene glycol) (PEG) hydrogel via thiol-ene photocrosslinking with non-degradable or proteolytically degradable peptide crosslinker. Hydrogel stiffness was manipulated by controlling the concentration of crosslinker. The metabolic activities and cytotoxicity of the encapsulated cells were measured by resazurin and Live/Dead assays, respectively. Cell harvesting was conducted via enzymatic degradation using α-chymotrypsin, and differentiation and maturation of the liberated DCs were evaluated by quantitative polymerase chain reaction and flow cytometry.

**Results:**

THP-1 cells well proliferated in the soft degradable hydrogel with a higher metabolic activity. However, the stiff matrix inhibited cell growth in 3D. The gene expression assay indicated that the 3D hydrogel condition was superior to 2D culture in terms of differentiation and maturation of DC. Interestingly, the stiffness of matrix was important factor in DC function. In the stiff hydrogel, the expression levels of differentiation and maturation markers were higher compared to the low stiffness hydrogel. The mature DCs caged in the hydrogel matrix were harvested after short enzymatic digestion of hydrogel and the liberated cells had over 90% viability. The flow cytometric result revealed that the proportion of CD80 + /CD86 + cells from the stiff hydrogel was relatively higher than cells either from 2D or soft hydrogel in 3D.

**Conclusion:**

The collected evidence indicated that the proteolytically degradable PEG hydrogel matrix promoted DC differentiation and maturation. In addition, the matrix stiffness control could manipulate the marker expressions of differentiation and maturation. Particularly, the mature DC was successfully collected from the hydrogel matrix. These results highlighted the PEG hydrogel-based DC culture might be a useful tool for potential DC-based immunotherapies.

**Supplementary Information:**

The online version contains supplementary material available at 10.1007/s10529-023-03457-w.

## Introduction

A dendritic cell (DC) is a representative antigen-presenting cell that plays a pivotal role in both innate and adaptive immunities (Eisenbarth 2019). DCs located in different organs (e.g., skin and intestine) recognize antigens and deliver the antigen information to T cells in lymph nodes (Worbs et al. 2017). In addition, DCs activate not only B cells but also natural killer cells (NK cells) by secreting cytokines (e.g., interferon [IFN], interleukin-12 [IL-12]), and interleukin-5 [IL-5]) (Lambotin et al. 2010). Malfunction or low activity of DC is closely related to various immune disorders, inflammatory diseases, and even cancers. For example, self-antigen recognition of DC is a crucial cause of diverse autoimmune diseases. Abnormal inflammatory responses, such as chronic inflammation, might be caused by prolonged cytokine expressions of DCs (Ganguly et al. 2013). In tumor growth, tumor cells evade the anti-cancer activity of DC by reducing the activity or accessibility of DCs (Wculek et al. 2020). Therefore, DCs are considered important target cells in various immunotherapies.

In most DC-targeted immunotherapies, activation and regulation of DC functions are critical methodologies, which can be conducted either in-vivo or in-vitro. For example, the in-vivo therapy can be achieved by delivering antigen-loaded nanoparticles or scaffolds to the DCs of a patient (Cheung et al. 2018; Liu et al. 2021). This method is quite simple and easily applied. Nonetheless, the low antigen delivery efficiency is a drawback and it is not suitable for DC-deficient patients (Le Gall et al. 2018; Zhu et al. 2020). In contrast, the DCs can be also activated in-vitro. In this method, the patient’s immune cells such as pre-DCs and monocytes are collected and subsequently cultured in-vitro. Then, the activated DCs are delivered to the patient (Tacken et al. 2007). In this case, the aforementioned low efficiency in antigen delivery can be solved with a sufficient number of DC.

Despite of several advantages, the in-vitro DC activation is still challenging in terms of therapeutic effect. Particularly for cancer patients, tumor cells and tissue inhibit the immune activity of dendritic cells through various pathways thus avoiding anti-cancer effect. The hypoxia of tumor microenvironment suppresses metabolic activity and viability of DCs (Taylor and Scholz 2022), or tumor cells reduce the immune activity of dendritic cells directly by secreting interleukin-6 (IL-6), interleukin-10 (IL-10), and transforming growth factor-β (TGF-β) (Wculek et al. 2020). In addition, tumor cells inhibit C–C motif chemokine receptor 7 (CCR7)-mediated migration of DCs. As a result, less than 5% of the total injected dendritic cells migrate to lymph nodes (de Vries et al. 2003). One of the ways to overcome these limitations is enhancing the DC activity by mimicking in-vivo condition (i.e., microenvironment and signaling molecules), in which monocytes and pre-DCs are differentiated and maturated in extracellular matrix-rich surroundings (Worbs et al. 2017). This is quite different from conventional DC culture method (i.e., suspension and tissue culture plate (TCP)-based culture), which does not recapitulate the physiological behavior of DC. Recently, several studies supported that differentiation and maturation of DC largely depend on the surrounding microenvironment. For instance, DCs cultured in a collagen matrix showed higher CD11c expression than those cultured on TCP (Sapudom et al. 2020). In addition, the expression levels of MHCII, CD86, and CD83 varied depending on crosslinking method of collagen matrix (Molzer et al. 2019). However, to our knowledge, a systemic comparison of DC differentiation and maturation in conventional and 3D matrix-based cultures has not been yet reported.

Poly(ethylene glycol) (PEG) is a biocompatible and bio-inert polymer that is widely used in medicine and biomaterial fabrication. For decades, PEG-based hydrogels have been explored as a 3D culture matrix (Cao et al. 2021; Kraehenbuehl et al. 2008; Lampe et al. 2010; Nam et al. 2019). The PEG-based hydrogels confer not only easy control of the viscoelastic property, but the integration of diverse bioactive materials in the hydrogel network (Fernandez-Yague et al. 2022). In particular, click-chemistry (e.g., thiol-norbornene reaction) enables a precise control of the network with extremely low toxicity, which is a great advantage in cell encapsulation (Lin et al. 2015). The PEG hydrogels crosslinked via thiol-ene photocrosslinking has been applied for various tissue engineering. β-cell spheroids encapsulated in PEG hydrogels successfully secreted insulin and chondrogenesis was promoted in the PEG hydrogel formed with matrix metalloproteinase (MMP)-sensitive linkers (Lin et al. 2011; Sridhar et al. 2015). Macrophage-like RAW264.7 cells in PEG hydrogels secreted more tumor necrosis factor α (TNF-α) than those cultured in 2D (Kim et al. 2019).

In this work, we hypothesized that a 3D microenvironment similar to soft tissue would affect the differentiation and maturation of DCs. First, we compared the DCs behavior between conventional 2D culture and 3D culture methods. We also evaluated how hydrogel properties (i.e., degradability and modulus) affected differentiation and maturation behavior in a 3D culture environment using PEG hydrogels. Additionally, we harvested the mature DC (mDC) cultured in PEG hydrogels via enzymatic degradation and confirmed whether the mDC maintained their immune activities.

## Materials and methods

### Materials

4-arm polyethylene glycol (PEG4OH) was purchased from JenKem Technology (Plano, TX, USA). Anhydrous dichloromethane (DCM) and ethyl ether were purchased from JT Baker (Phillipsburg, NJ, USA) and Thermo Fisher Scientific (Waltham, MA, USA), respectively. 5-norbornene-2-methylamine was obtained from Tokyo Chemical Industry (Tokyo, Japan) and used without further purification. Lyophilized α-chymotrypsin was purchased from VMR Life Science (Randor, PA, USA). The protease-sensitive peptide crosslinker (H_2_N-KCGPLGLYAGCK-amide) was obtained via the custom peptide synthesis service of Biostem (Suwon, Korea). Unless otherwise noted, all other chemicals were purchased from Sigma-Aldrich (St. Louis, MO, USA).

### Synthesis

PEG-tetra-urethane-norbornene (PEG4uNB) was synthesized according to an established protocol (Park et al. 2011). Briefly, the vacuum-dried PEG4OH (20 kDa) was dissolved in DCM at 20% (w/v) with 4-(dimethylamino) pyridine (DMAP) (0.5 equivalent molar amount of the hydroxyl group of PEG4OH) and trimethylamine (TEA) (three equivalent molar amounts). After 15-min stirring, five equivalent molar amounts of 4-nitrophenyl chloroformate (PNC) were added dropwise to the PEG solution in an ice bath. The reaction was allowed to proceed at room temperature (RT) under nitrogen gas for 24 h. The product was precipitated in cold ethyl ether, followed by vacuum drying. The dried PEG4PNC was redissolved in DCM at 20% (w/v) in a round bottom flask, and five equivalent molar amounts of 5-norbornene-2-methylamine were slowly added into the solution using a dripping funnel. The reaction proceeded at RT under nitrogen gas for 6 h, and the resulting PEG4NB was precipitated in cold ethyl ether, followed by vacuum drying. For purification, the dried PEG4NB was redissolved in deionized water and dialyzed for three days against deionized water using a cellulose acetate tube (MWCO: 12–14 kDa), followed by lyophilization.

### Hydrogel fabrication

For acellular hydrogel preparation, a hydrogel precursor solution was prepared by dissolving PEG4uNB, photoinitiator (lithium arylphosphinate [LAP]), and dithiol crosslinker in pH 7.4 phosphate buffered saline (PBS). As dithiol crosslinker, 1,4-dithiothreitol (DTT) or a protease-sensitive peptide (KCGPLGLYAGCK) was used in non-degradable or proteolytically degradable hydrogel formation, respectively. The concentration of dithiol crosslinker for each hydrogel type was shown in Supplementary Table 2, while the concentrations of PEG4NB and LAP were fixed at 5 wt% and 1 mM, respectively. The precursor solution was injected between glass slides separated by 1 mm-thick spacer, and irradiated by UV light (365 nm, 5 mW/cm^2^) for 2 min. Shear modulus of hydrogel was measured by a rotational rheometer (HAKKE MARSIII, Thermo Fisher Scientific) after 24-h incubation in PBS at 37 °C to achieve equilibrium swelling. The hydrogel slab was punched out by a biopsy punch of 8 mm in diameter and measured in an oscillatory strain-sweep mode (0.1–5%) under a nominal force of 0.2–0.3 N and a gap of 0.8 mm with a parallel plate geometry (diameter: 8 mm). The shear storage modulus (G′) was determined by averaging the measured modulus values in the linear viscoelastic region. An enzymatic degradation profile was obtained by measuring the residual mass ratio of hydrogel after incubation in α-chymotrypsin solution (1 mg/mL) at 37 °C in PBS (pH 7.4). The swollen hydrogel mass was measured after removing excess water from the surface with a paper wiper.

1 $$Residual \, mass \, ratio = Hydrogel \, mass \, after \, enzyme \, treatment/Initial \, hydrogel \, mass$$

### Cell culture

Human monocyte THP-1 cells were maintained in Roswell Park Memorial Institute 1640 Medium (Corning, Corning, NY, USA) containing 10% (v/v) fetal bovine serum (Gibco, Waltham, MA, USA), 1% (v/v) antibiotic–antimycotic (Gibco) and 0.05 mM 2-mercaptoethanol and incubated at 37 °C and 5% CO_2_. The culture medium was replaced every 3–4 days. For cell encapsulation, the PEG hydrogel precursor solution was filtered with a 0.2 μm pore syringe filter and THP-1 cells were suspended in the solution at 2 × 10^6^ cells/mL. 20 μL of the cell-contained precursor solution was injected into cylindrical mold (diameter: 5 mm) and exposed to UV light (365 nm, 5 mW/cm^2^) for 2 min. After UV irradiation, the resulting cell-laden hydrogel was 5 mm in diameter and 1 mm in thickness and immediately immersed in culture media. For differentiation to THP-1-derived immature dendritic cells (iDC), suspended (1.5 × 10^5^ cell/mL) or encapsulated THP-1 cells (40,000 cell/gel) were incubated with Granulocyte–macrophage colony-stimulating factor (GM-CSF) (100 ng/mL; Woongbee Meditech, Seoul, Korea) and interleukin-4 (IL-4) (100 ng/mL; Woongbee Meditech, Seoul, Korea) in the growth medium for 6 days, and maturation of the iDC was induced by culturing in 1 μg/mL of lipopolysaccharide (LPS) in the growth medium for 24 h (T. Y. Li and Chiang 2019).

Metabolic activity of encapsulated cells was measured by resazurin assay. Resazurin (Sigma-Aldrich) was dissolved in PBS at 2.5 mg/mL and filtered using a 0.2 μm pore syringe filter. Then, the resazurin solution was then diluted 100 times with serum-free culture medium. The cell-laden hydrogel was incubated in 500 μL of the diluted resazurin solution at 37 °C and 5% CO_2_ for 4 h. As a reference sample (control), the diluted resazurin solution was incubated with no cells at same condition for 4 h. After incubation, 200 μL of the solution was transferred to a 96-well plate and the fluorescence level was measured in arbitrary unit (AU) using a microplate reader (excitation: 560 nm; emission: 590 nm; Synergy HT; BioTek, Winooski, VT, USA). For live/dead staining, cell-laden hydrogels were incubated in PBS containing calcein AM (1 μM) and ethidium homodimer-1 (4 μM) for 1 h, and then washed with PBS. The hydrogels were observed under a fluorescent microscope (CELENA S; Logos Biosystems, Anyang, Korea) and the acquired images were stacked (100 μm thick; 10 μm per slice).

### Cell harvesting

After culturing THP-1-derived dendritic cells in PEG hydrogels, the cells were harvested via enzymatic degradation of cell-laden hydrogels. The hydrogels were immersed in α-chymotrypsin solution (1 mg/mL, 400 μL/gel) at 37 °C, and escaped cells were collected by centrifugation. The viability of obtained cells was assessed by trypan blue exclusion assay.

### Reverse-transcription-quantification polymerase chain reaction

Suspended cells or cell-laden hydrogels were collected and frozen with liquid nitrogen for gene expression analysis. RNA extraction was performed using a AccuPrep Universal RNA extraction kit (Bioneer, Daejeon, Korea). Single-stranded cDNA was prepared by converting from isolated RNA using PrimeScript RT reagent kit (TaKaRa, Kyoto, Japan). Quantitative real-time polymerase chain reaction (PCR) was performed using the SYBR Premix Ex Taq II kit (TaKaRa) and a Quantstudio 1 real-time PCR machine (Applied Biosystems, Forster City, CA, USA). Samples were run at 95 °C for 30 s, followed by 40 cycles of 95 °C for 5 s, 55 °C for 30 s, and 72 °C for 30 s. Amplification of the SYBR signal was detected at the end of each cycle. The expression levels of target genes were normalized to that of GAPDH (internal control) using the 2^–ΔΔCT^ method. The primer sequences were listed in Supplementary Table 1.

### Flow cytometry

THP-1-derived dendritic cells were collected and washed with PBS. Then, the cells were fixed with 4% (w/v) paraformaldehyde for 10 min and washed three times with PBS. After fixation, the cells were blocked in Tris-buffered saline with 1% (w/v) bovine serum albumin (BSA) for 30 min at RT. Then, the cells were stained with recommended dilution of fluorescent dye-conjugated monoclonal antibodies (FITC anti-human CD80 and APC anti-human CD86 antibodies; Biolegend, San Diego, CA, USA) for 30 min at RT and washed with PBS containing 0.05% (v/v) Tween 20 (Bio-Rad; Herculles, CA, USA). The fluorescence levels were measured using a flow cytometer (BD Accuri; Becton Dickinson, Franklin Lakes, NJ, USA) and the obtained data were analyzed and plotted by BD Accuri C6 software. Prior to obtaining a fluorescence density plot, forward and side scattering were measured and at least 10,000 cells were gated based on both scattering parameters (SSC-A; FSC-A) to reject dead cell debris. As a control group, undifferentiated monocyte cultured in 2D was measured. The measurement was triplicated and the representative data were shown in Fig. 4.

### Statistics

All experiments were performed in triplicate, and the data are presented as mean ± standard deviation (SD). The statistical analysis was conducted by GraphPad Prism 9. For between-group comparison, the Student’s t-test was performed based on the results of the Shapiro–Wilk normality test and the F-test. For multiple group comparisons, one-way analysis of variance with the Bonferroni post hoc test was performed. P-values * < 0.05, ** < 0.01, and *** < 0.001 were considered statistically significant.

## Results and discussion

**Fig. 1:**
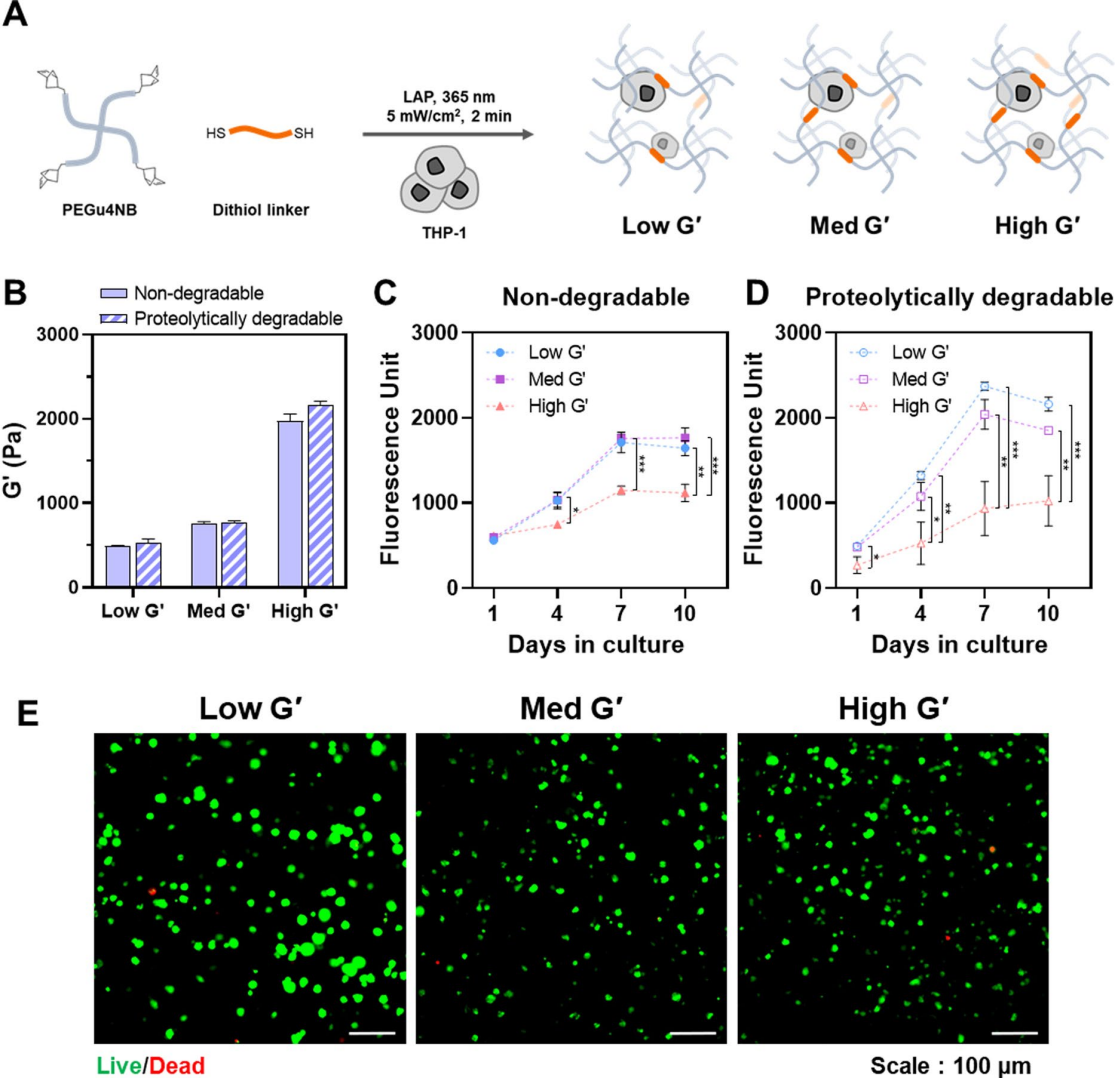
Encapsulation and 3D culture of THP-1 monocytes in PEG hydrogels of different stiffness. **A** Schematic of THP-1 encapsulation in PEG hydrogels via thiol-ene photocrosslinking. The shear storage modulus was modulated by controlling the crosslinker concentration. **B** Shear storage moduli of PEG hydrogels formed with non-degradable and proteolytically degradable crosslinkers (n = 3, mean ± SD). **C** & **D** Relative metabolic activity of THP-1 monocytes cultured in PEG hydrogels formed of different degradability and moduli for 10 days: **C** Non-degradable crosslinker (DTT); **D** Proteolytically degradable linker (n = 3, mean ± SD). Statistical significance level was shown as *, **, and *** for P < 0.05, < 0.01, and < 0.001, respectively. (E) Live/dead fluorescence images of THP-1 monocytes cultured in proteolytically degradable PEG hydrogels of different moduli on day 10 post-encapsulation. (scale bar = 100 μm, green: live; red: dead)

Figure 1A shows the encapsulation of THP-1 cells in thiol-norbornene PEG hydrogels via photocrosslinking. The network was formed by orthogonal click-chemistry, which provided easy control of crosslink density by varying stoichiometric ratio between norbornene group and thiol group of crosslinker. Herein, the shear storage modulus was modulated in the range of 500–2000 Pa by adjusting the dithiol crosslinker concentration (5–8 mM) with a fixed PEG4uNB concentration (5 wt%) (Fig. 1B and Supplementary Table 2 and Fig. 1). According to shear storage modulus, the PEG hydrogels were designated as low G′ (500 Pa), med G′ (800 Pa), and high G′ (2000 Pa). Specifically, the moduli of low and med G′ hydrogels corresponded to shear moduli of normal tissues of breast, liver, or kidney (Chaudhuri et al. 2020; Guimaraes et al. 2020). By contrast, the high G′ hydrogel mimicked abnormal tissues stiffened by fibrosis in tumor or chronic inflammation (Martinez-Vidal et al. 2021).

Figure 1C and 1D show the metabolic activity changes of THP-1 cells encapsulated in hydrogels formed with different crosslinker types and concentrations. Regardless of hydrogel stiffness, the metabolic activity increased until day 7, indicating the encapsulated cells proliferated in hydrogels. Passing day 7, the metabolic activity reached the plateau that was caused by crowding effect (Chang et al. 2019). As expected, the metabolic activity of high G′ group was relatively lower than those of the other group for both ‘non-degradable’ and ‘proteolytically degradable’ gels, because the dense network restrained cell division (Banerjee et al. 2009). For low and med G′ groups, proteolytically degradable hydrogels revealed higher metabolic activities than those of non-degradable hydrogels, indicating that degradable network was suitable for cell growth. The peptide crosslinker used in formation of the degradable hydrogels was sensitive against various MMPs (Ki et al. 2014). Hence, the reside cells could remodel the microenvironment, facilitating proliferation and migration (Wei et al. 2020). In contrast, the effect of cell binding ligand (i.e., RGDS) was not significant on cell growth in the hydrogel matrix (Supplementary Fig. 2). We speculated that non-adherent type THP-1 cells were less affected by the binding ligand.

Figure 1E shows live/dead fluorescence images of THP-1 cells cultured in the proteolytically degradable hydrogels of different stiffness on day 10 post-encapsulation. The encapsulated cells were mostly viable, regardless of matrix stiffness. It means that even high G′ hydrogel network did not restrict the material diffusion for cell survival. Particularly, the cells in the low G′ hydrogel formed distinctly larger clusters than those in the other hydrogels. Similar to previous reports, the encapsulated cells could have enough space for cell division in the loose network of the low G′ hydrogel (Sun et al. 2017), while the high G′ hydrogel restrained the large cluster formation due to a high crosslink density.

**Fig. 2:**
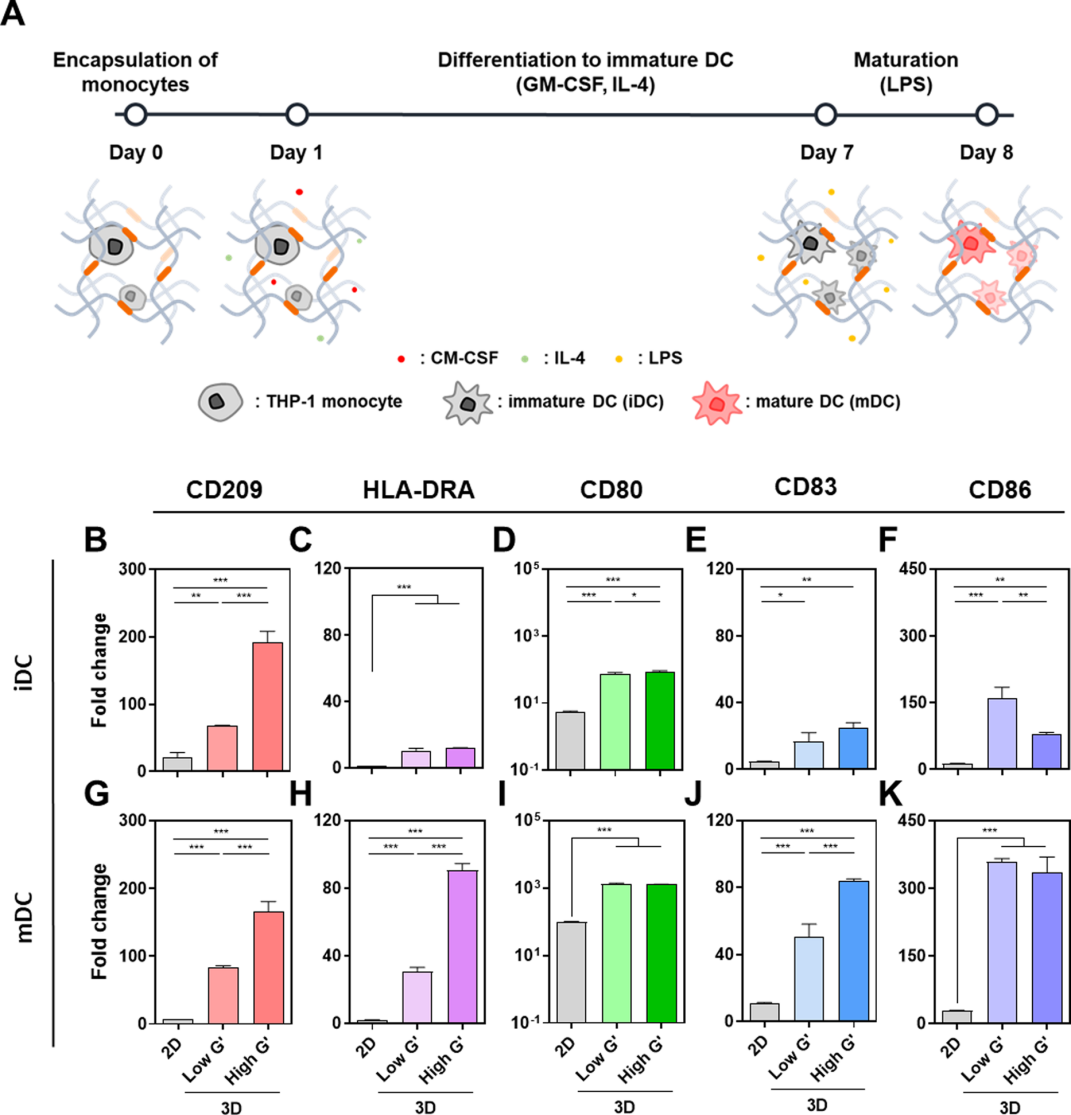
Differentiation and maturation of THP-1-derived dendritic cells in proteolytically degradable PEG hydrogels of different stiffness. **A** Process of maturation of THP-1-derived dendritic cells in 3D. **B**–**K** Relative mRNA expression levels of DC differentiation and maturation gene markers of THP-1-derived iDCs and mDCs in PEG hydrogels of different stiffness: (B) CD209, **C** HLA-DRA, (D) CD80, **E** CD83, and **F** CD86 of THP-1-derived iDCs; **G** CD209, **H** HLA-DRA, **I** CD80, **J** CD83, and **K** CD86 of THP-1-derived mDCs. One-fold indicates the expression level of each gene of THP-1 monocyte (undifferentiated) in 2D (n = 3, mean ± SD). Statistical significance level was shown as *, **, and *** for P < 0.05, < 0.01, and < 0.001, respectively

Based on these observations, the effect of matrix stiffness on DC maturation was evaluated using the proteolytically degradable low and high G′ hydrogels, because they showed obvious difference in terms of cell growth. As shown in Fig. 2A, the encapsulated THP-1 monocytes were differentiated by GM-CSF and IL4 treatment and subsequently matured with LPS in the PEG hydrogel matrices of different stiffness. We first analyzed metabolic activity changes during the differentiation of DCs. The metabolic activities during differentiation showed similar behavior to those with growth medium, showing a steady increase over culture period (Supplementary Fig. 3). In particular, in the low G′ hydrogel, the highest metabolic activity on day 7 and 8 might be caused by promoted metabolism during differentiation and maturation of DC. The mitochondrial biogenesis, a process of increasing the number of mitochondria, was driven by upregulation of peroxisome proliferator-activated receptor γ (PPARγ) and PPARγ co-activator 1α (PCG1α) during DC differentiation. DC maturation also promoted the respiratory reserve capacity and fatty acid synthesis through glycolysis and pentose phosphate pathway (Pearce and Everts 2015). In contrast, we could not observe a meaningful increment in metabolic activity in the high G′ group during differentiation and maturation. It was caused by the low number of cells in the stiff hydrogel as well as spatial restriction of dense network.

Next, the mRNA expression levels of gene markers associated with differentiation and maturation of DC were analyzed. Figure 2B–F show the relative expression levels of THP-1-derived iDCs prepared in different culture conditions. After differentiation, all genes were upregulated compared with those of THP-1 monocytes (undifferentiated) in 2D and the upregulated gene expression levels were significantly higher in 3D hydrogel matrices. Interestingly, the expression level of CD209, a C-type lectin receptor on DC surface, was exceptionally higher in the High G′ hydrogel compared with the low G′ hydrogel (Fig. 2B). It suggests that the stiff microenvironment facilitated the DC differentiation and the iDCs in the high G′ hydrogel were likely to recognize antigen molecules (van Kooyk and Geijtenbeek 2003).

After LPS treatment, the marker expressions were further upregulated except CD209 (Fig. 2G–K), which indicates iDCs were matured successfully in the 3D condition regardless of matrix stiffness. In particular, the expression levels of HLA-DRA and CD83 were higher in the high G′ hydrogel than those in the low G′ hydrogel. HLA-DRA plays a key role in delivering antigens to T cells and was affected by matrix stiffness (Reith et al. 2005). CD83 is a protein expressed in mDCs and regulates maturation, activation, and homeostasis of DC. It also stabilizes MHCII, thereby inducing the activation of effective T cell (Li et al. 2019). Previous study reported that the DC differentiation was influenced by substrate property via mechanosensory signals. Stiff and elastic hydrogels induced DC differentiation and maturation from monocytes via the PI3K-γ signaling pathway which upregulates integrin binding and subsequent cortical F-actin assembly (Vining et al. 2022). In addition, the stiff substrate promoted proliferation, differentiation, and maturation of DCs with upregulation of pro-inflammatory cytokines (i.e., IL6 and TNF-α) via a TAZ-mediated Hippo-signaling pathway (Chakraborty et al. 2021). By contrast, Sapudom et al. reported an opposite observation that DCs were less differentiated and matured in 3D collagen matrix than on the tissue culture plate. However, such a result might be due to too low stiffness (Young’s modulus of = 46.94–219.44 Pa) of the collagen matrix (Sapudom et al. 2020). Taken together, our observation indicated that the stiff 3D matrix was effective for DC differentiation as well as maturation.

**Fig. 3:**
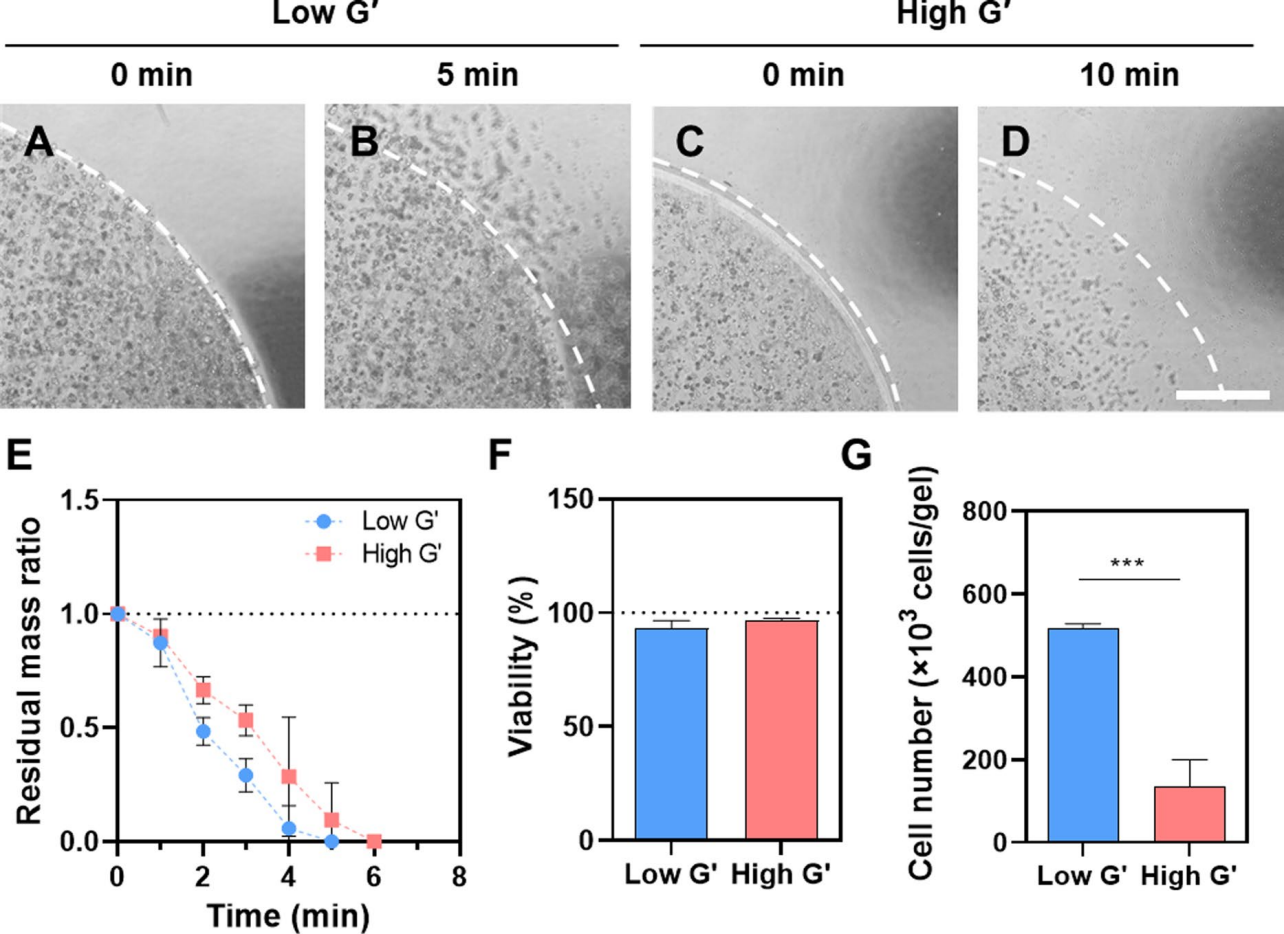
Harvesting of THP-1-derived mDCs encapsulated in proteolytically degradable PEG hydrogels of different stiffness. The cells were liberated by α-chymotrypsin treatment (1 mg/mL). **A** & **B** Bright-field images of low G′ cell-laden hydrogel degradation at 0 and 5-min enzyme treatment; **C** & **D** High G′ cell-laden hydrogel degradation at 0 and 10-min enzyme treatment, respectively. Scale bar = 200 μm. **E** Residual mass ratio changes of low and high G′ hydrogels during α-chymotrypsin treatment (n = 3, mean ± SD). **F** Viabilities of cells liberated from low and high G′ hydrogels (n = 3, mean ± SD). **G** Harvested live cell quantities from a single gel after 8-day culture (n = 3, mean ± SD). Statistical significance level was shown as *, **, and *** for P < 0.05, < 0.01, and < 0.001, respectively

The encapsulated cells were readily collected by enzymatic degradation of hydrogels. The MMP-sensitive crosslinker (KCGPLGLYAGCK) was sensitive against α-chymotrypsin and the hydrogel was therefore eroded quickly by the enzyme treatment (Ki et al. 2014). As shown in Fig. 3A–D, we observed distinct surface erosion in both low and high G′ hydrogels within 10 min, resulting in cell liberation. Figure 3E shows the degradation kinetics of low and high G′ hydrogels in α-chymotrypsin-contained PBS. Both gels were rapidly degraded and no trace of hydrogel was seen after 6 min. Of course, the high G′ gel was degraded relatively slowly. It was caused by a low diffusion rate of enzyme as well as the dense network (Shie et al. 2020; Skaalure et al. 2016). Based on the degradation kinetics, we set the optimal time for α-chymotrypsin treatment as 5 and 10 min for low and high G′ hydrogels, respectively and checked the viability of the liberated cells from the hydrogel matrix. Figure 3F and 3G present the cell viability and the collected number of cells from low and high G′ hydrogels. The liberated cells showed a viability of over 90% regardless of the hydrogel stiffness, which indicated that the cell harvesting process including enzyme treatment and washing procedure was not harmful to mDCs. The collected cell numbers were nearly 500,000 and 130,000 cells per gel for low and high G′ hydrogels, respectively (Fig. 3G). The harvested cell numbers were 12.5- and 3.25-fold greater in low and high G′ hydrogels, respectively, compared to the initial cell number (40,000 cells per gel). Such a difference in live cell number was consistent with the former metabolic activity measurement (Fig. 1D). Therefore, this result confirmed that the longer enzyme treatment for high G′ hydrogel did not cause further cell loss in the harvesting process.

**Fig. 4:**
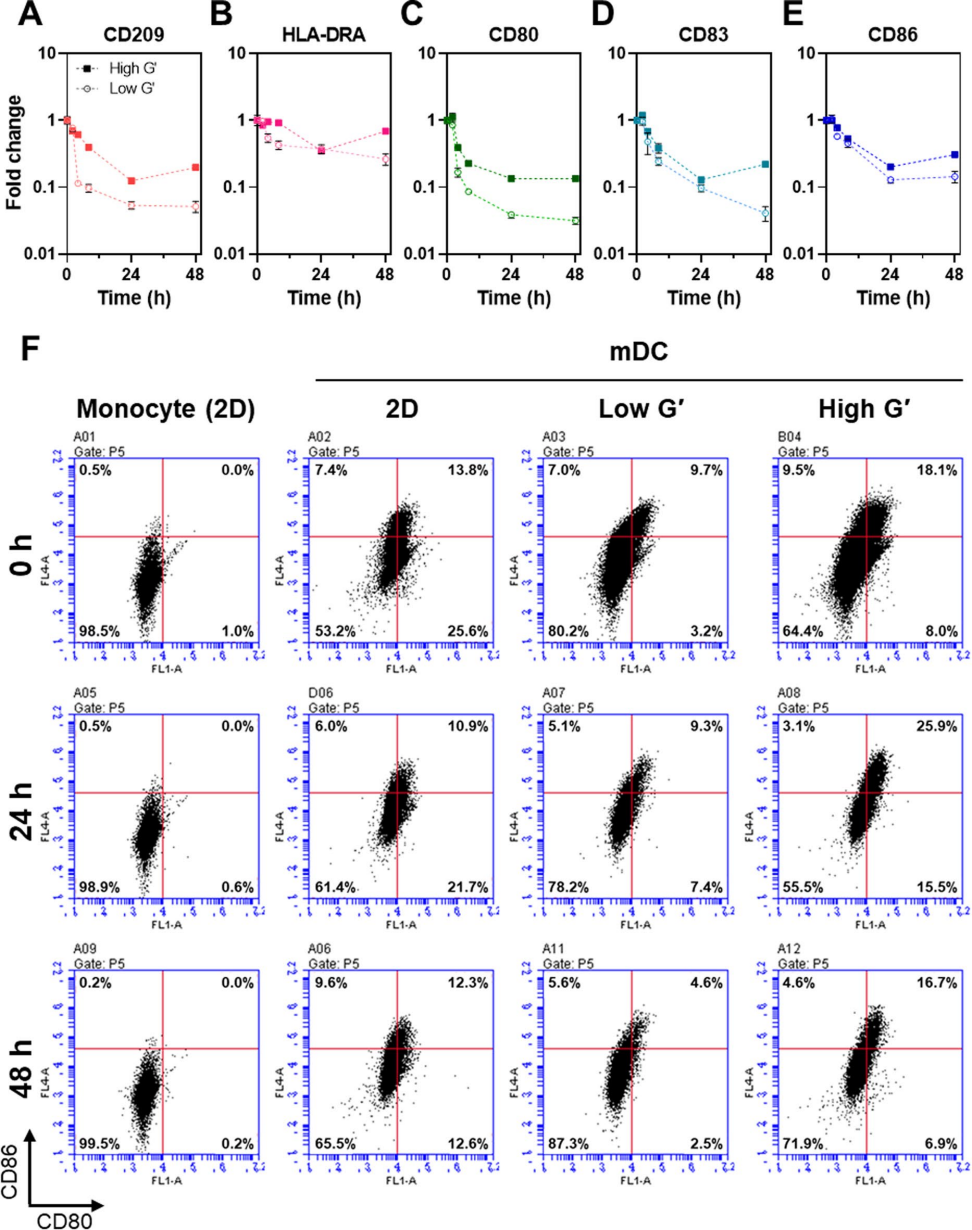
Analysis of DC marker expressions of harvested mDCs from hydrogels of different stiffness. **A**–**E** Time-dependent change in relative mRNA expression levels of THP-1-derived mDCs after harvesting from Low G′ and High G′ hydrogels: **A** CD209, **B** HLA-DRA, **C** CD80, **D** CD83, and **E** CD86. One-fold is the expression level immediately after harvesting (0 h). Solid and open symbols indicated gene expression of mDCs in the high G′ and low G′ hydrogels, respectively (n = 3, mean ± SD). (F) Flow cytometry results of relative CD80 and CD86 expressions in THP-1 monocytes and mDCs that were prepared in different culture conditions (2D, 3D-Low G′, and 3D-High G′). The percentage of CD80- and CD86-positive cells were displayed at the upper-right corner

Next, we evaluated how long the DC activity was maintained after harvesting. It is crucial for DC-based immunotherapy, because the delivery efficiency of mDC to a lymph node depends on the activation level and duration (Choi et al. 2022; Worbs et al. 2017). In the previous report, the injected DC was found in lymph nodes of melanoma patients 12–18 h after injection and lost its motility within 48 h (de Vries et al. 2003). Hence, we assessed the DC marker expression changes during 48 h after cell harvesting. Figure 4A–E show that all gene expression levels decreased rapidly after harvesting except HLA-DRA, regardless of hydrogel stiffness. Nevertheless, the depression in the relative gene expression levels of mDCs from the high G′ hydrogel was higher than that of the low G′ hydrogel for 48 h. The activation level of the harvested mDCs was further analyzed at the protein level (i.e., CD80 and CD 86) using flow cytometry. In Fig. 4F, mDCs collected from 2D TCP and PEG hydrogels kept the elevated expression levels of CD80 and CD86 until 48 h compared with monocytes (undifferentiated), while the mRNA expression levels of the related genes decreased sharply after harvesting (Fig. 4C and 4E). In addition, the flow cytometry result showed the higher activation level of mDCs collected from the high G′ hydrogel compared with cells from 2D or low G′ hydrogel. At 24 h, the percentage of CD80^+^/CD86^+^ cells from the high G′ hydrogel was 25.9%, while the values of 2D and low G′ hydrogel were 10.9% and 9.3%, respectively. These results strongly support that the stiff 3D matrix contributed to DC maturation and the collected mDC might have the high migration activity. Our observations imply that the microenvironment is another crucial factor in regulating DC property, while previous studies have focused on immune active material-based DC activation (Ding et al. 2019; Fang et al. 2017; Miller et al. 2005). We speculate the contribution of the hydrogel matrix to the DC activation was closely associated with mechanical stress on the cytoskeleton of DC by the surrounding polymer network. However, the low influence of RGDS motif tethered on the network does not support this speculation, because most studies explained that mechanosensing is initiated from integrin-binding on the substrate (Kechagia et al. 2019). It might be explained by the high motility of DC, which is less dependent on adhesion, unlike other anchorage-dependent cells. However, the exact relationship between DC activation and microenvironment should be further investigated in the future study.

## Conclusion

The tailored PEG hydrogel was suitable for culture, differentiation, and maturation of DC in 3D. The hydrogel matrix might provide the encapsulated cells with a proper microenvironment similar to in-vivo conditions. In 3D, relatively lower stiffness and proteolytically degradable matrix facilitated cell proliferation. In contrast, the high G′ hydrogel showed better performance in differentiation as well as maturation. In addition, we showed that a quick and low-cytotoxic enzyme treatment liberated the activated DC from the PEG hydrogel, and the harvested DCs retained their immune activities for 48 h, which might be sufficient for DC delivery to lymph nodes in practical therapy. These findings imply that the hydrogel-based DC culture is a suitable platform to improve the efficacy of DC-based immunotherapies.

### Supplementary Information

Below is the link to the electronic supplementary material.

Supplementary file1 (DOCX 84 kb)
