# Supplementary material for: Differentiation, maturation, and collection of THP-1-derived dendritic cells based on a PEG hydrogel culture platform
Source: Biotechnol Lett. 2024 Jan 17;46(2):235–47. doi: 10.1007/s10529-023-03457-w (PMC10901936; doi:10.1007/s10529-023-03457-w)
Supplement: Supplementary file 1 — Supplementary file1 (DOCX 84 kb) [file 10529_2023_3457_MOESM1_ESM.docx]

**Supplementary Table 1** RT-qPCR primer sequences for THP-1-derived dendritic cells.

| Genes | Primer sequence (5'-3') | Ref. |
| --- | --- | --- |
| GAPDH | F-AGCCTCAAGATCATCAGCAATG | (Wu et al. 2017) |
|  | R-CACGATACCAAAGTTGTCATGGAT |  |
| CD209 | F-AAATCAGGAAGGCACGTGGCAATG | (Gustafsson et al. 2008) |
|  | R-TGTTGGGCTCTCCTCTGTTCCAAT |  |
| HLA-DRA | F-TCGAAATGGAAAACCTGTCACC | (Yamagami et al. 2006) |
|  | R-CCCAATAATGATGCCCACCA |  |
| CD80 | F-TGGTGCTGGCTGGTCTTTC | (Son et al. 2019) |
|  | R-CGTTGCCACTTCTTTCACTTCC |  |
| CD83 | F-AGGTTCCCTACACGGTCTCC | (Sanarico et al. 2011) |
|  | R-TTGCAGCTGGTAGTGTTTCG |  |
| CD86 | F-GGGCCGCACAAGTTTTGA | (Duan et al. 2019) |
|  | R-GCCCTTGTCCTTGATCTGAA |  |

**Supplementary Table 2** Crosslinker compositions of non-degradable and proteolytically degradable PEG hydrogels of different stiffness (i.e., low G′, med G′, and high G′) and their shear storage moduli.

|  | DTT | | Proteolytically degradable linker  (KCGPLGLYAGCK) | |
| --- | --- | --- | --- | --- |
|  | [SH] (mM) | G′ (Pa) | [SH] (mM) | G′ (Pa) |
| Low G′ | 5.5 | 493.8 ± 3.2 | 5.5 | 527.0 ± 43.1 |
| Med G′ | 6.5 | 753.4 ± 26.1 | 6.0 | 774.7 ± 13.1 |
| High G′ | 8.0 | 1976.9 ± 80.6 | 7.5 | 1946.0 ± 19.1 |

**Supplementary Figure 1**


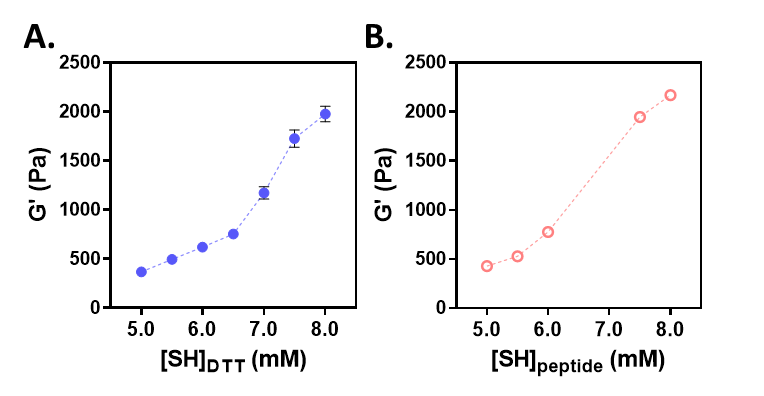


**Supplementary Figure 1** Shear storage moduli of PEG hydrogels with varying concentration of crosslinker in the prepolymer solution. (A) Non-degradable linker (DTT). (B) Proteolytically degradable peptide linker (n = 3, mean ± SD).

**Supplementary Figure 2**


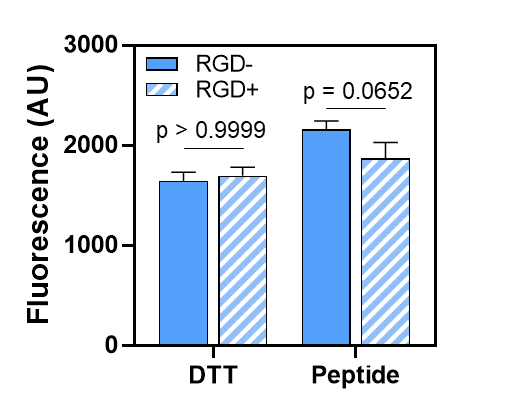


**Supplementary Figure 2** Relative metabolic activity of THP-1 cells encapsulated in PEG hydrogels formed with or without CRGDS peptide (2 mM) (n = 3, mean ± SD).

**Supplementary Figure 3**


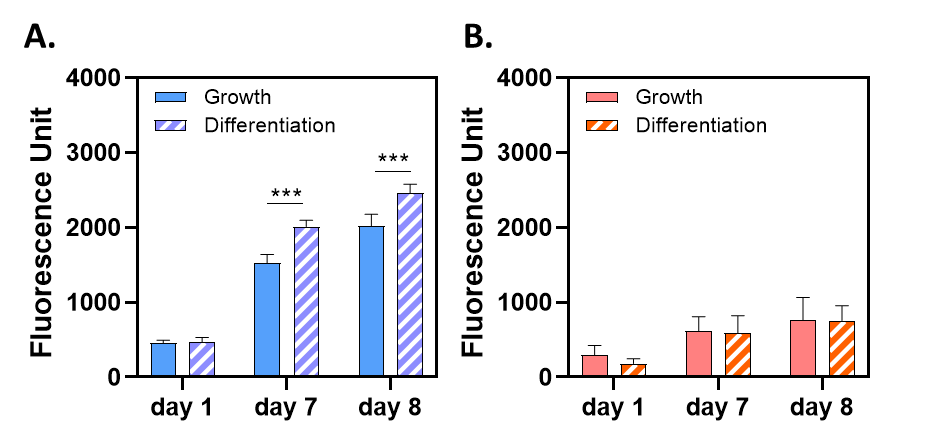


**Supplementary Figure 3** Relative metabolic activity of THP-1-derived DCs encapsulated in PEG hydrogels of (A) low G′ and (B) high G′ (n = 3, mean ± SD). *** indicates the statistical significance level at P < 0.001.

**Lists of References in supplementary materials**

Duan XQ, Liu X, Li WT, Holmes JA, Kruger AJ, Yang CH, Li YJ, Xu M, Ye HY, Li S, Liao XZ, Sheng QJ, Chen D, Shao T, Cheng ZM, Kaj B, Schaefer EA, Li SL, Chen LM, Lin WY, Chung RT (2019) Microrna-130a downregulates hcv replication through an atg5-dependent autophagy pathway. Cells-Basel 8(4):338. https://doi.org/10.3390/cells8040338.

Gustafsson C, Mjosberg J, Matussek A, Geffers R, Matthiesen L, Berg G, Sharma S, Buer J, Ernerudh J (2008) Gene expression profiling of human decidual macrophages: Evidence for immunosuppressive phenotype. Plos One 3(4): e2078. https://doi.org/10.1371/journal.pone.0002078.

Sanarico N, Colone A, Grassi M, Speranza V, Giovannini D, Ciaramella A, Colizzi V, Mariani F (2011) Different transcriptional profiles of human monocyte-derived dendritic cells infected with distinct strains of mycobacterium tuberculosis and mycobacterium bovis bacillus calmette-guerin. Clin Dev Immunol 2011:741051 https://doi.org/10.1155/2011/741051.

Son Y, Choi J, Kim B, Park YC, Eo SK, Cho HR, Bae SS, Kim CD, Kim K (2019) Cyclosporin a inhibits differentiation and activation of monocytic cells induced by 27-hydroxycholesterol. Int Immunopharmacol 69:358–367. https://doi.org/10.1016/j.intimp.2019.01.045.

Wu YT, Jin FJ, Liu JW, Zheng DL, Wang YL, Wu FY, Zhu YX, Wang YF (2017) Protective effect of theaflavin-3-digallate on lipopolysaccharide-induced inflammation injury in macrophage cells. Int J Pharmacol 13(8):980–989. https://doi.org/10.3923/ijp.2017.980.989.

Yamagami S, Ebihara N, Usui T, Yokoo S, Amano SO (2006) Bone marrow-derived cells in normal human corneal stroma. Arch Ophthalmol-Chic 124(1):62–69. https://doi.org/10.1001/archopht.124.1.62.
